# Serum Levels of IL-10 and IL-17A in Occult HBV-Infected South-East Iranian Patients

**Published:** 2010-03-01

**Authors:** Mohammad Kazemi Arababadi, Ali Akbar Pourfathollah, Abdollah Jafarzadeh, Gholamhossein Hassanshahi

**Affiliations:** 1 Department of Microbiology, Hematology and Immunology, Faculty of Medicine,Rafsanjan University of Medical Sciences, Rafsanjan, Iran; 2 Molecular Medicine Research Center, Rafsanjan University of Medical Sciences, Rafsanjan, Iran; 3 Department of Immunology, School of Medical Sciences, Tarbiat Modares University, Tehran, Iran

**Keywords:** Occult Hepatitis B Infection, Interleukin-10, Interleukin-17, HBV-DNA

## Abstract

**Background and Aims:**

Occult hepatitis B infected (OBI) patients can not completely eradicate hepatitis B virus-DNA (HBV-DNA) from their liver and peripheral blood. The main aim of this study was to investigate the Interleukin (IL)-10and IL-17A serum levels in patients suffering from OBI.

**Material and Methods:**

In this observational study, plasma samples of 3700 blood donors were tested for hepatitis Bsurface antigen (HBsAg) and antibodies to the hepatitis B core antigen (anti-HBc), using enzyme-linked immunosorbent assay (ELISA). The HBsAg-/anti-HBc+ samples were selected and screened for HBV-DNA, using the polymerase chain reaction (PCR). HBV-DNA positive samples were assigned as OBI cases and IL-10 and IL-17 serum levels were detected using ELISA.

**Results:**

The results demonstrated that, 352 (9.5%) out of 3700 blood samples were HBsAg-/anti-HBc+ and HBV-DNA was detected in 57/352 (16.1%) of the HBsAg-/anti-HBc+ samples. Our results showed that the IL-10 and IL-17A serum levels increased significantly in the OBI cases in comparison to the controls (P < 0.001).

**Conclusions:**

According to the results of this study the higher level of IL-10 production may suppress the functioning of the immune system against HBV in OBI patients. The elevated IL-17A serum level also indicates a long period of infection in the patients observed.

## Introduction

Occult hepatitis B virus infection (OBI) is a clinical form of hepatitis B which in spite of an undetectable amount of the hepatitis B surface antigen (HBsAg) in the patient's serum, the hepatitis B virus (HBV) DNA is obviously visible in the patient's serum [1][2][3]. This type of hepatitis is one of the main challenges for blood transfusion services and even if all of the donated blood and blood components are screened for HBsAg, some cases of post-transfusion hepatitis B have been reported [4]. In most instances, the main cause of post transfusion hepatitis B infection is OBI [5], which we found in our previous investigations in Isfahan and Kerman, the two central provinces of Iran [5][6]. However, the mechanism(s) responsible for the progression of OBI have yet to be clarified but some investigators have suggested the key roles of genetic and immunological parameters in the resistance of some individuals as well as the susceptibility of others [7][8].

In the immune system, cytokines play an important role in appropriate immune response to viral infections [9]. Interleukin (IL)-10 is the main cytokine in the regulation of cellular immunity against viral infections, which is produced by T helper 2 (TH2) and T regulatory lymphocytes [10]. IL-10 has potent inhibitory effects on some parts of the immune system, especially on cell-mediated immune responses [10]. Therefore, alterations in this cytokine enable the immune system to reduce its eligible immune response against viral infections [10][11]. Previous studies showed that IL-17A plays a crucial role in chronic infection by means of several mechanisms [12][13]. For example, IL-17A upregulates anti-apoptosis molecules in hepatocytes [12], and in fact, increases the survival of the HBV reservoir. IL-17A also prevents target cell destruction by means of cell-mediated immunity [12]. Accordingly, the aim of this study was to examine the IL-10 and IL-17A serum levels in OBI patients in order to find out the mechanism(s) involved in the pathogenesis of the disorder.

## Materials And Methods

### Subjects

Peripheral blood samples were collected from 3700 volunteer blood donors at the Rafsanjan Blood Transfusion Services, Kerman, Iran in ethylenediaminetetraacetic acid (EDTA) pre-coated 5.5 ml tubes. The samples were centrifuged at 370×g for 4 minutes. All of the sera were separated within 24 hours following their collection. If necessary, serum samples were stored at -20ºC for a maximum of 2 months or at -70ºC (for longer periods) for further use. For the analysis of polymorphisms, a 2 ml sample was collected from patients with OBI (57 cases) and from one hundred healthy controls (HBsAg-/HBV-DNA-/ antibodies to the hepatitis B core antigen positive [anti-HBc+]). This study was approved by the ethical committee of Rafsanjan University of Medical Sciences

NB: all of the participants in this study filled out and signed the informed consent form which was designed with the aims and objectives of the study in mind.

### Detection of serological HBV markers 

The HBsAg screening tests were performed by enzyme-linked immunosorbent assay (ELISA) (Behring, Germany). The antibodies to the hepatitis B core antigen (anti-HBc) screening test was also performed by a manual microplate enzyme immunoassay, using a commercial anti-HBc kit (RADIM, Italy). The present method is based on a competitive enzyme immunoassay (EIA). All of the samples were also screened by using ELISA (RADIM, Italy) for possible hepatitis C virus (HCV) and human immunodeficiency virus (HIV) infections.

### HBV- DNA Extraction from plasma samples 

Viral DNA was purified from 200 μl of plasma samples. Briefly, each plasma sample was incubated at 72ºC for 10 minutes and then cooled down to 4ºC for 5 minutes in 200μl proteinase K (200 μg/ml). Following phenol/chloroform extraction (1:1), the viral DNA was precipitated with ethanol and the pellet was re-dissolved in DNase free, deionized water and stored at -20ºC for further use.

### HBV-DNA PCR and Gel Electrophoresis

To amplify the S gene of HBV-DNA a PCR reaction mixture was made by the addition of the following reagents to a 0.2 ml micocentrifuge tube on ice: 2.5 µl of Taq DNA polymerase buffer (10x), 0.5 µl of MgCl2 (a stock concentration 1.5 mM), 0.5 µl of each dNTP [(dATP, dCTP, dGTP, dTTP) a stock concentration of 10 mM], 1 µl of each primer pair (stock concentration of 25 ng/µl), 5 µl of prepared DNA and sterile double distilled water to a final volume of 25 µl. The sequence of primers was as follows: F: 5`-TCGTGGTGGACTTCTCTC-3` and R: 5`-ACAGTGGGGGAAAGCCC-3` The PCR thermocycler was run with the following program: one cycle at 93°C for 2 mins, 93°C for 1 min (denaturation), 1 min at 55°C (annealing), 72°C for 40 sec (elongation) followed by 30 cycles at 93ºC for 20 sec, 55ºC for 20 sec and 72ºC for 40 sec. During the last 45 seconds of the first stage, 0.3 µl of Taq DNA polymerase was added to the mixture

The HBV genome was purchased from Cinnagen-Iran and used as the positive control. The PCR product (10 µl) was run on a 2% agarose gel after adding 4 µl of loading buffer. The presence of a 500bp fragment indicated a positive result.Parallel to the samples, a 100bp DNA ladder was also run to estimate the molecular size of the DNA fragments. The current technique can detect 100 copies per microliter.

### Detection of the cytokine serum level

The IL-10 and IL-17A serum levels were measured using ELISA (eBioscience, ESP) in both patients and healthy controls, immediately after blood collection. Assays were performed according to the manufacturer's guidelines. The sensitivity of kits was 2 pg/ml and the coefficient variation (CV) for inter- or intra-assay was found to confirm the assessment reliability.

### Liver enzymes evaluation

serum glutamic-pyruvic transaminase (SGPT), serum glutamic oxaloacetic transaminase (SGOT), direct and total bilirobin and alkaline phosphatase (ALP) of OBI patients were measured using MAN Ltd kits, Iran.

### Statistical analysis

The differences in variables were analyzed by student t-tests. P values of less than 0.05 were considered significant.

## Results

This study was performed on 3700 collected blood samples at the Rafsanjan Blood Transfusion Services. All of the samples were found to be negative for HBsAg, antibodies to HCV (anti-HCV), and antibodies to HIV (anti-HIV). Out of 3700 samples, 352 (9.5%) cases were found to be positive for anti-HBc; HBV-DNA was detected in 57/352 (16.1% of HBsAg negative but anti-HBc positive) of them. Results of this study indicated that the HBV-DNA was detectable in 16.1 % of HBsAg negative but anti-HBc positive samples collected, which is 1.54% (57/3700) of the total collected samples.

Our results showed that there was no significant difference between groups with regard to age, sex and socio-economic condition Table 1. Socio-economic condition was measured by level of education and by monthly income. All of the samples were also negative for HIV and HCV antibodies.

**Table 1:** Demographic and socioeconomic conditions of occult hepatitis B virus-infected patient and controls.

**Variant**	**Halethy Control**	**Patient**	**P-value**
**Age**	28 ± 8	28 ± 6	P > 0.3
**Sex**			
**Female**	3 (3%)	2 (3.5%)	P > 0.2
**Male**	97 (97.8%)	55 (96.5%)	P > 0.19
**Socio-Economic Status**			
**Weak**	22 (22%)	12 (21%)	P > 0.1
**Medium**	47 (47%)	28 (49%)	P > 0.1
**High**	31 (31%)	17 (30%)	P > 0.1

We found that the IL-10 serum level was 15.05 ± 1.1 pg/ml and 10.2 ± 1.0 pg/ml in OBI patients and in the healthy controls, respectively. Statistical analysis showed that there was a significant difference between the two groups (P < 0.001).

We also showed that there was a significant increase in the IL-17A serum level (12.48 ± 2.00 pg/ml) in OBI patients in comparison to healthy controls (4.43 ± 0.54 pg/ml). Statistical analysis showed a significant difference between the two groups (P < 0.001).

The serum levels of ALT, AST, direct and total bilirubin were 24U/L, 30U/L, 0.2 mg/dl and 1.2 mg/dl, respectively, while the serum level of ALP was 23 U/L, SGOT was 28 U/L, direct bilirubin was 0.15 mg/dl and total bilirubin was 1.1 mg/dl in healthy controls. Statistical analysis showed that there was no significant difference between the two groups with reference to the above parameters (P > 0. 1).

## Discussion

It has been reported that in chronic viral infections, the pattern of cytokine expression may have some important modifications [14]. It is not well defined why OBI patients are unable to completely overcome viral contamination.However, it seems that cytokines play a key role in the clearance of HBV; and several studies indicated that NK cells and cytotoxic T cells (the two important cells in cellular immunity) depend on cytokine balance to function at their best. [15][16]. Therefore, this study was aimed at determining the IL-10 serum level as a cytokine suppressor of cellular immunity and IL-17A as a chronic disease inducer. Our results showed that the IL-10 and IL-17A serum levels are significantly higher in OBI patients than in the controls. Therefore, based on our results it can be concluded that a higher IL-10 level can suppress immune responses to HBV infection in OBI patients. On the other hand, IL-17A upregulates anti-apoptosis molecules in hepatocytes [12], thus leading to increased longevity of the HBV reservoir. To our knowledge, this is the first study that has been carried out to evaluate the serum levels of these cytokines in OBI patients. However, in a study, Bozkaya H et al., showed that there was a significant increase in the IL-10 serum level in chronic HBV infection among the Turkish population [10]. An elevated production of IL-10 by dendritic cells in chronic HBV infected patients has also been reported by Wang et al., [17]. Fan XG et al., also showed a significant increase in the IL-10 serum level in chronic HCV infection [18].On the other hand, our previous study showed that the IL-12 serum level did not increase in patients with OBI [19]; thus it can be concluded that these patients are unable to express a sufficient concentration of IL-12 for HBV clearance and this may be related to the antagonistic effects of IL-10 in these patients. Interestingly, the previous study showed that patients with chronic HBV infection suffer from defective functioning of the dendritic cells, which may be associated with the inability of efficient presentation of HBV antigens to the host immune system for the eradication of HBV [17]. This property of dendritic cells in chronic HBV infection probably is associated with an elevated IL-10 level. Xu Y et al., have reported that IL-17A protein and mRNA levels were increased in liver injuries following chronic HBV infection, cirrhosis and hepatocarcinoma [13]. In agreement with our study, an elevated IL-17 A serum level in HBV infected patients was also shown by Qin LY and colleagues [20]. Other studies have also demonstrated that there was an increase in the IL-6 serum level as an inducer of Th17 clonal expansion in HBV-infected patients [21][22][23]. However, some studies have reported an enhanced level of IL-17A in other viral infections [24][25] but as far as we know this is the first reported in OBI. Investigators are suggesting that the circulating cytokine profile in HBV infection is related to the replication level of the virus and to the activity of liver disease. Based on this concept that the HBV replication level is very low and liver disease is relatively silent in OBI patients, it probably could be concluded that the immune system tends to be unresponsive to HBV. The IL-10 serum level may increase to suppress immune responses to HBV, while an elevated IL-17A level causes increased hepatocyte survival.

Finally, due to the complexity of OBI, other aspects of the disease need to be examined and a recommended future task for investigators would be to investigate cytokine expression and the polymorphisms of other important related cytokines and their receptors, at the level of both protein and mRNA in OBI patients.
